# Increased Incidence of Atrial Fibrillation in Patients with Rheumatoid Arthritis

**DOI:** 10.1155/2015/809514

**Published:** 2015-03-01

**Authors:** A. Kirstin Bacani, Cynthia S. Crowson, Véronique L. Roger, Sherine E. Gabriel, Eric L. Matteson

**Affiliations:** ^1^Division of Rheumatology, Department of Internal Medicine, Mayo Clinic, Rochester, MN 55905, USA; ^2^Department of Health Sciences Research, Mayo Clinic, Rochester, MN 55905, USA; ^3^Division of Cardiology, Department of Internal Medicine, Mayo Clinic, Rochester, MN 55905, USA

## Abstract

*Objective*. To investigate the incidence of atrial fibrillation (AF) among patients with rheumatoid arthritis (RA) compared to the general population. *Methods*. A population-based inception cohort of Olmsted County, Minnesota, residents with incident RA in 1980–2007 and a cohort of non-RA subjects from the same population base were assembled and followed until 12/31/2008. The occurrence of AF was ascertained by medical record review. *Results*. The study included 813 patients with RA and 813 non-RA subjects (mean age 55.9 (SD:15.7) years, 68% women in both cohorts). The prevalence of AF was similar in the RA and non-RA cohorts at RA incidence/index date (4% versus 3%; *P* = 0.51). The cumulative incidence of AF during follow-up was higher among patients with RA compared to non-RA subjects (18.3% versus 16.3% at 20 years; *P* = 0.048). This difference persisted after adjustment for age, sex, calendar year, smoking, and hypertension (hazard ratio: 1.46; 95% CI: 1.07, 2.00). There was no evidence of a differential impact of AF on mortality in patients with RA compared to non-RA subjects (hazard ratio 2.5 versus 2.8; interaction *P* = 0.31). *Conclusion*. The incidence of AF is increased in patients with RA, even after adjustment for AF risk factors. AF related mortality risk did not differ between patients with and without RA.

## 1. Introduction

Rheumatoid arthritis (RA) is a systemic inflammatory autoimmune disease associated with an increased risk of cardiovascular disease and premature mortality [1, 2]. The primary research focus in vascular disease in patients with RA has been on coronary heart disease. Recent publications have examined the relationship between atrial fibrillation (AF) and RA with variable results [3, 4]. We examined the incidence, prevalence, and mortality impact of AF in a population-based inception cohort of patients with RA and a comparator population from the same community.

## 2. Methods

### 2.1. Study Population

The population of Olmsted County, Minnesota, is well-suited for investigation of the epidemiology of RA and AF because comprehensive medical records on all residents are available through a record linkage system. The potential of this data system for use in population-based studies has been described [5].

An inception cohort of all cases of RA diagnosed between January 1, 1980, and December 31, 2007 (*n* = 813), among Olmsted County residents ≥18 years of age was assembled as described [6]. Incidence date was defined as the earliest date at which the patient fulfilled at least 4 of the 7 American College of Rheumatology 1987 classification criteria for RA [7]. All cases were followed up longitudinally through their entire medical records until death, migration, or 12/31/2008. A comparison cohort of Olmsted County residents without RA with similar age, sex, and calendar year was identified. The index date for each non-RA subject was defined as the RA incidence date of the corresponding patient with RA.

The medical records of each cohort were electronically crossmatched with a database of electrocardiogram (ECG) data. Since all ECG data were obtained during clinical care, it was not available for all patients or at specified intervals. AF was defined as the date that AF was first noted on an ECG. Cardiovascular risk factor and outcome data have been collected in both cohorts as described [8] including cigarette smoking status (current, former, or never); presence of dyslipidemia, hypertension, or diabetes mellitus; personal history of coronary heart disease (presence of angina pectoris, coronary artery disease, myocardial infarction, or coronary revascularization procedures (e.g., coronary artery bypass graft or angioplasty)); height and weight measurements at baseline and computed body mass index (BMI); and family history of coronary heart disease (defined as presence of coronary heart disease in first-degree relatives at age <65 years for females and <55 years for males). Outcomes included mortality, coronary heart disease (as defined for personal history), and heart failure (defined by Framingham criteria) [9].

### 2.2. Statistical Analysis

Descriptive statistics (percentages, mean, etc.) were used to summarize the characteristics of each cohort and comparisons between cohorts were performed using Chi-square and rank sum tests. The cumulative incidence of AF adjusted for the competing risk of death was estimated [10]. Patients with AF prior to RA incidence/index date were removed from these analyses because they were not at risk of developing AF. Cox proportional hazard models were used to examine the association between potential risk factors and the development of AF. Dichotomous time-dependent covariates were used to represent risk factors that developed during follow-up; patients were considered unexposed until the time when the risk factor developed and then they changed to exposed. A sensitivity analysis was performed to examine the possibility that differences in ECG testing rates might influence cumulative incidence results. A subset of ECG tests was randomly selected for patients with RA to mimic the testing rate in patients without RA for the sensitivity analysis.

## 3. Results

The study population consisted of 813 patients with RA and 813 subjects without RA. There were 556 (68%) women, and the mean age (SD) at RA incidence/index date was 55.9 (15.7) years. The average length of follow-up was 9.6 (6.9) years among the patients with RA and 10.9 (7.2) years among the non-RA subjects. Cardiovascular risk factors at RA incidence date/index date were similar in both cohorts except for a higher prevalence of smokers among the RA patients compared to the non-RA subjects (*P* = 0.002; Table 1).

**Figure 1:**
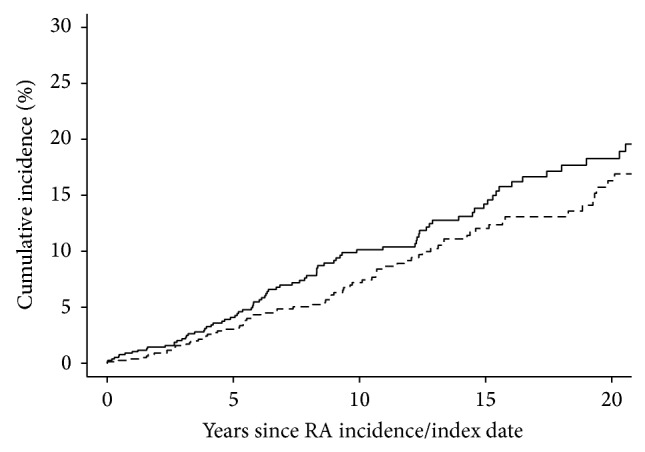
Cumulative incidence of atrial fibrillation in patients with rheumatoid arthritis (RA; solid line) compared to non-RA subjects (dashed line), prevalent atrial fibrillation removed (*P* = 0.048).

There was no difference in the prevalence of AF at RA incidence/index date among patients with RA compared to non-RA subjects (number, %) (*n* = 33, 4% versus *n* = 28, 3%), *P* = 0.51. During follow-up, 89 patients with RA and 73 non-RA subjects developed AF. The cumulative incidence of AF during follow-up was marginally higher among patients with RA (18.3% at 20 years; 95% confidence interval (CI): 14.2, 22.3) compared to non-RA subjects (16.3% at 20 years; 95% CI: 12.3, 20.2; *P* = 0.048; Figure 1). This difference corresponded to a hazard ratio (HR) of 1.60 (95% CI: 1.17, 2.18) adjusted for age, sex, and calendar year of RA incidence/index date. This difference persisted after additional adjustment for current smoking status and development of hypertension (HR: 1.46; 95% CI: 1.07, 2.00). Additional potential risk factors for development of AF in patients with RA are summarized in Table 2.

Across calendar year of follow-up, the rate of AF increased among non-RA subjects (6% per year; *P* = 0.002 adjusted for age and sex). Among patients with RA, the increase in AF was less pronounced (3% per year; *P* = 0.070). However, there was no statistically significant difference between trends (*P* = 0.20 for interaction between calendar year of follow-up and RA/non-RA). The rate of ECG testing decreased over calendar time for both cohorts. The RA cohort had a consistently higher rate of ECG testing compared to the non-RA cohort (77.9 ECGs per 100 person-years (py) in RA compared to 62.7 ECGs per 100 py in non-RA; rate ratio = 1.24; *P* < 0.001). This difference persisted when the analysis was limited to patients without cardiovascular disease (by censoring patients when they developed cardiovascular disease) (HR: 1.83; 95% CI: 1.20, 2.78). In a sensitivity analysis, when ECGs for patients with RA were randomly chosen to mimic the ECG testing rates in patients without RA, the number of RA patients with AF was only reduced by 3. This reduction resulted in a smaller difference in the cumulative incidence of AF between groups (17.9% in RA versus 16.3% in non-RA at 20 years; *P* = 0.088); however, the age, sex, and calendar year adjusted results still demonstrated a significant increase in AF among the RA compared to the non-RA (HR: 1.59; 95% CI: 1.16, 2.18).

During follow-up, 229 patients with RA and 163 non-RA subjects died. AF was associated with mortality in both cohorts (HR 2.5; 95% CI: 1.8, 3.3 in RA and HR 2.8; 95% CI: 1.9, 4.0 in non-RA). There was no evidence that AF had a different impact on mortality among patients with RA compared to non-RA subjects (interaction *P* = 0.31). Similarly, there was no evidence that AF exerted a differential effect on the development of coronary heart disease (interaction *P* = 0.79) or heart failure (interaction *P* = 0.66) in patients with RA compared to non-RA subjects.

## 4. Discussion

Our study examined the occurrence of AF in a systematic manner in a population-based cohort of RA patients and found that the prevalence of AF was not different among patients with RA compared to non-RA subjects. The cumulative incidence of AF was increased in patients with RA compared to non-RA subjects. Over the observation period, the incidence of AF increased among non-RA subjects and increased to a lesser extent among patients with RA. Finally, AF was not more strongly associated with mortality in patients with RA than in patients without RA.

Lindhardsen et al. reported the incidence of AF and stroke among patients with RA in a Danish national registry [3] identifying patients by ICD codes, prescription data, and hospital admission data. The study reported an overall 40% higher incidence of AF in patients with RA compared to the general population and did not adjust for all cardiovascular risk factors. We found that patients with RA have a slightly increased risk of developing AF that persists after adjusting for smoking and hypertension.

Kim et al. reported an increased incidence of hospitalization for AF in patients with RA compared to non-RA patients using data from a commercial insurance plan [4]. After adjusting for risk factors including diabetes, cardiovascular disease, medications, and healthcare utilization, the risk of AF was no longer increased among patients with RA compared to non-RA subjects and compared to patients with osteoarthritis.

Coronary heart disease is a known contributor to the excess mortality experienced by patients with RA. In the general population, coronary heart disease is a risk factor for development of AF, and AF is an independent predictor of increased mortality [11]. Our study accounted for cardiovascular risk factors and found that AF was equally associated with mortality both in patients with RA and non-RA subjects. Chronic inflammation may play a role in the development of AF [12–14]; some of our findings may reflect this possibility. A potential association between diastolic dysfunction and AF has been described, and patients with RA are known to have increased prevalence of diastolic dysfunction [13, 14]. Other identified potential risk factors for the development of AF among patients with RA include severe extra-articular RA, multiple sedimentation rates >60 mm/hour, and use of cox-2 inhibitors.

Strengths of our study include our population-based incident cohort comprised of patients who fulfill standardized criteria for RA, the long-term follow-up, and the record linkage system which permits capture of nearly all of the cases of RA in the community and minimizes referral bias. Occurrence of AF was recorded based on a cardiologist's interpretation of electrocardiogram data and included both inpatient and outpatient occurrences of AF.

Potential limitations of our study include that it was retrospective and that the population of Olmsted County, Minnesota, is predominantly Caucasian; however, the results are in general applicable to other patient populations [15]. Also, our data collection method did not enable categorizing AF into paroxysmal, persistent, or permanent or excluding postoperative AF.

## 5. Conclusions

In addition to the well-known increased risk for coronary heart disease in patients with RA, these patients also have an increased risk for AF. The mechanisms underlying this risk, including possibly diastolic dysfunction, require further elucidation.

## Figures and Tables

**Table 1:** Characteristics of 813 patients with rheumatoid arthritis (RA) and 813 subjects without RA.

Characteristic	RA (*n* = 813)	Non-RA (*n* = 813)	*P* value
Age at incidence/index, years, mean ± SD	55.9 ± 15.7	55.9 ± 15.7	0.9
Sex, female, *n* (%)	556 (68%)	556 (68%)	0.9
Race, white, *n* (%)	761 (94%)	771 (96%)	0.1
Body mass index at incidence/index, kg/m^2^, mean ± SD	27.8 ± 6.0	27.7 ± 6.0	0.8
Smoking status at incidence/index, *n* (%)			0.002
Never	364 (45%)	435 (54%)	
Current	178 (22%)	144 (18%)	
Former	271 (33%)	234 (29%)	
Diabetes mellitus at incidence/index, *n* (%)	79 (10%)	67 (8%)	0.3
Hypertension at incidence/index, *n* (%)	307 (38%)	275 (34%)	0.1
Coronary heart disease at incidence/index, *n* (%)	87 (11%)	89 (11%)	0.9
Heart failure at incidence/index, *n* (%)	23 (3%)	23 (3%)	0.9
Length of follow-up, years, mean ± SD	9.6 ± 6.9	10.9 ± 7.2	—

**Table 2:** Risk factors for atrial fibrillation at rheumatoid arthritis (RA) incidence among 813 patients with RA in 1980–2007.

Characteristic	Hazard ratio (95% CI)
At rheumatoid arthritis incidence	
ESR at RA incidence, per 10 mm/hour increase	1.10 (0.99, 1.22)
Rheumatoid factor positive	1.35 (0.88, 2.08)
Current smoker	1.29 (0.78, 2.13)
Former smoker	1.00 (0.63, 1.57)
Family history of coronary heart disease	1.16 (0.72, 1.89)
At rheumatoid arthritis incidence or during follow-up (time-dependent)	
Comorbid condition	
Hypertension	2.01 (0.72, 5.59)
Diabetes mellitus	1.39 (0.86, 2.24)
Dyslipidemia	0.77 (0.48, 1.25)
Coronary heart disease	1.53 (0.96, 2.45)
Obesity (BMI ≥ 30 kg/m^2^)	1.33 (0.87, 2.04)
Underweight (BMI < 20 kg/m^2^)	1.51 (0.88, 2.58)
Alcohol abuse	1.87 (0.92, 3.81)
Rheumatoid arthritis disease characteristics	
Rheumatoid nodules	1.42 (0.91, 2.22)
Erosive or destructive RA	1.29 (0.84, 1.98)
Severe extra-articular RA^*^	**3.29 (1.98, 5.48)**
Large joint swelling	1.48 (0.85, 2.57)
ESR > 60 mm/hr on 3 occasions	**2.04 (1.19, 3.50)**
Major joint arthroplasty	1.45 (0.92, 2.26)
Medications	
Methotrexate	1.46 (0.93, 2.28)
Hydroxychloroquine	0.91 (0.59, 1.41)
Other DMARDs^**^	1.35 (0.83, 2.18)
Biologic agent	1.34 (0.57, 3.15)
Corticosteroids	1.40 (0.87, 2.27)
Cox-2 inhibitor	**1.73 (1.10, 2.73)**
ASA (≥6 tabs/day for ≥3 mo)	1.03 (0.63, 1.70)
NSAID	0.96 (0.49, 1.90)

^*^Pericarditis, pleuritis, Felty's syndrome, major cutaneous or other organ vasculitis, neuropathy, scleritis, episcleritis, retinal vasculitis, or glomerulonephritis.

^**^Disease modifying antirheumatic drug includes gold, sulfasalazine, azathioprine, cyclophosphamide, cyclosporine, D-penicillamine, or leflunomide.

ASA: acetylated salicylates; BMI: body mass index; CI: confidence interval; ESR: erythrocyte sedimentation rate (Westergren); NSAID: nonsteroidal anti-inflammatory drug.
